# Self-Protected Virtual Sensor Network for Microcontroller Fault Detection

**DOI:** 10.3390/s22020454

**Published:** 2022-01-07

**Authors:** German Sternharz, Jonas Skackauskas, Ayman Elhalwagy, Anthony J. Grichnik, Tatiana Kalganova, Md Nazmul Huda

**Affiliations:** 1 Department of Electronic and Electrical Engineering, College of Engineering, Design and Physical Sciences, Brunel University London, Uxbridge UB8 3PH, UK; german.sternharz@brunel.ac.uk (G.S.); jonas.skackauskas2@brunel.ac.uk (J.S.); tatiana.kalganova@brunel.ac.uk (T.K.); mdnazmul.huda@brunel.ac.uk (M.N.H.); 2 Blue Roof Labs, 759 N Main St., Eureka, IL 61530-1035, USA; Tony.Grichnik@BlueRoofLabs.com

**Keywords:** Virtual Sensor Network, Digital Twin, Mahalanobis Distance, Neural Network, LSTM, uncertainty estimation, cybersecurity, Industrial Control System

## Abstract

This paper introduces a procedure to compare the functional behaviour of individual units of electronic hardware of the same type. The primary use case for this method is to estimate the functional integrity of an unknown device unit based on the behaviour of a known and proven reference unit. This method is based on the so-called virtual sensor network (VSN) approach, where the output quantity of a physical sensor measurement is replicated by a virtual model output. In the present study, this approach is extended to model the functional behaviour of electronic hardware by a neural network (NN) with Long-Short-Term-Memory (LSTM) layers to encapsulate potential time-dependence of the signals. The proposed method is illustrated and validated on measurements from a remote-controlled drone, which is operated with two variants of controller hardware: a reference controller unit and a malfunctioning counterpart. It is demonstrated that the presented approach successfully identifies and describes the unexpected behaviour of the test device. In the presented case study, the model outputs a signal sample prediction in 0.14 ms and achieves a reconstruction accuracy of the validation data with a root mean square error (RMSE) below 0.04 relative to the data range. In addition, three self-protection features (multidimensional boundary-check, Mahalanobis distance, auxiliary autoencoder NN) are introduced to gauge the certainty of the VSN model output.

## 1. Introduction

Rapid development of electronic hardware is in high demand due to the competitive market space, set product life cycles and customer demand [1]. At the same time, it is important to ensure that the manufactured hardware operates as intended and is tested in the development process based on prototypes. Otherwise, the result could lead to additional customer support, costly recalls or warranty claims.

For safety-critical applications, the requirement for functional integrity usually has implications on the health and safety of humans [2] or critical infrastructures of whole societies [3,4]. An unexpected breach of the functional integrity of electronic hardware can occur due to a multitude of reasons. Internal factors include errors in the manufacturing process, software bugs, defective components, upgrades or redesigns of hardware/software. Similarly, external influences can also interfere with the proper operation of electronic devices. This includes the use of new or changed components due to a changed supply from manufacturers or change of the supplier. Hardware degradation is another factor, which can cause avoidable costly failures or downtime [5,6]. Finally, malicious activities such as hacking attacks or sabotage should be considered [3,4]. The validation of proper functioning can greatly supplement the efforts to reduce risks and the impact of potential malicious activity.

The proposed method is based on the virtual sensor network (VSN) approach [7,8], which is adapted in this work to model the functional response behaviour of an electronic device based on the provided input signals. The electronic inputs and outputs of a proven reference device are measured and used for the training of a NN model for the VSN. Therefore, the model outputs the nominal desired response of the electronic device based on received inputs of an unknown device unit under test. When comparing the model output to the measured output of the unknown test device, deviations between the two can be identified. Moreover, upon detection, the specific differences in functionality can be described and further assessed, since the response behaviour of a device is both measured and modelled in a raw signal form. This also allows use of the virtual reference output as a fault-redundant signal source. Therefore, this approach has an advantage over classifiers or regression models, which provide a fault state or rating directly.

As with any model, there is a risk of model errors in the proposed method, which can lead to a deviation between a false model output and a properly functioning test device. Therefore, to avoid misinterpretation, conditions where the model is not qualified to provide a correct output due to model errors should be distinguished from conditions where the model indicates actual significant differences between the input/output relation of reference and test device measurements. In this study, this is achieved by self-protection features, which aim to gauge the uncertainty of the resulting model output. For this purpose, the observed multidimensional input sample of a monitored test device is compared to the space spanned by the training data inputs of the reference model. The following three self-protection features are presented in this study:

- A binary indicator of the uncertainty by a hypercube boundary-check.
- A prediction uncertainty measure based on the Mahalanobis distance (MD).
- An auxiliary autoencoder NN, which reconstructs the input signals to detect input anomalies during operation/testing compared to previously trained input or environmental conditions.

To demonstrate the proposed VSN method (including self-protection) for the identification of functional integrity and to validate it on actual hardware measurements, a remote-controlled drone is used. An unmodified drone controller is used as the reference device providing the desired functional behaviour of the drone, whereas another hardware controller with modified firmware simulates a controller fault or acts as a malicious test device. It is shown that faulty output of the modified controller is successfully detected by the proposed method. In addition, the self-protection features are applied to the outputs of an intentionally deficient VSN model with high model errors due to insufficient training of the underlying NN. It is demonstrated that the implemented self-protection features successfully indicate false predictions of the deficient VSN model.

The presented methodology was initially developed by the authors of this paper in frame of the AFWERX Microelectronics Supply Chain Provenance Challenge organised by the US Air Force Research Labs. The team consisting of the industrial partner Supply Dynamics and academic authors of this paper demonstrated this technology and was declared winner of the competition at the 50th International Test Conference (ITC), which took place in Washington, D.C., in November 2019 [9]. The contributions of the present work can be summarized as follows:

- A novel extension of the VSN method is introduced, which models the functional reference behaviour of electronic hardware to validate or diagnose the functional integrity of unknown device units.
- Operational measurements from a drone are gathered during nominal and malfunctioning controller conditions and used to validate the proposed method and illustrate its application.
- Three self-protection features of increasing complexity are applied and evaluated side-by-side.

Hereafter, this paper is structured as follows: Section 2 contains a literature review, providing context of the proposed VSN method and relevance of the foundational NN technology. Section 3 focuses on the proposed methodology and is subdivided into sections covering the measurement acquisition, the applied NN model and self-protection features. This is followed by a demonstration and validation of the introduced methods on remote-controlled drone hardware in Section 4. Finally, results from the drone experiment are discussed and conclusions on the conducted study are drawn with an outlook on potential future work in Section 5 and Section 6, respectively.

## 2. Literature Review

This section investigates the origin of virtual sensing as a subtopic of digital twin (DT) technology and the reasons for its increasing popularity in industrial and commercial use. Related research in the field of cybersecurity is evaluated in comparison to the proposed approach. The different methods of modelling sensors and systems are also explored and compared and the advantages of NNs for use in this context is justified. Different types of NNs suitable for long-term dependency learning are additionally further discussed. Examples of the effective use of these NNs as well as their drawbacks are looked into, in order to justify the methodology and NN model used.

### 2.1. Digital Twin Technology

A DT can be defined as a virtual instance of a system or process that is used to model its operation for the purpose of inferring details relating to the system, such as the status of its current operation or a prediction into its future operation. It has been noted by many [10,11,12,13,14,15,16] that this process is integral to the automation and optimisation of industrial processes, commonly referred to as Industry 4.0. Whilst the general concept was introduced in a presentation in the early 2000s under a different name [13], a practical implementation was first explored in 2012 by NASA [14], where the usage of existing physical models and sensor data to forecast vehicle health and remaining useful life was proposed. Subsequently, various implementations for the realisation of DTs were proposed and successfully demonstrated in a variety of use cases. For instance, an optimisation of the speed and efficiency of a production line using a DT model was proposed by Vachálek et al. [11]. The researchers were able to iterate various parameters in the virtual instance of their production line to gain insight into the dynamics of the process and using this data, they were able to successfully reduce the production time without interfering with the physical model during their testing.

An example of DT technology’s use on a smaller scale is demonstrated in [15], where a DT of a physical DC-DC power converter was created for the purpose of condition monitoring without the addition of extra circuitry. The authors opted to use linearised versions of the differential equations of the buck converter as opposed to calculating the eigenvalues and eigenvectors of the differential equations, due to the heavy computational demand of the latter. However, it was noted that this method reduces the accuracy of the model. While the proposed method was able to successfully provide a reasonable estimation of the parameters, as well as successfully monitor the degradation of the MOSFET and capacitor, there was a clear issue with the accuracy of the model which could be put down to the linearisation of the differential equation.

These papers, as well as others exploring similar methodology, exhibit the limitations with accuracy and reliability when creating DTs using linear models as well as the computational complexity in implementing a more accurate model using traditional methods.

A detailed review of existing literature on more advanced DT research based on AI technology is found for example in [16]. The work introduces a distinction between three types of DTs based on the degree of data flow automation between the DT and the represented physical system. It subdivides existing literature in the categories healthcare, smart cities and manufacturing with further subcategories. Another review study [1] explains the demand for DT technology for development and manufacturing from the point of view of a competitive market environment. It presents the state of current software tools for virtual validation of electronic hardware related to thermal characteristics, mechanical stress, and electromagnetic compatibility.

Electronic hardware platforms are acknowledged as integral components of Internet of Things (IoT) systems in [16], and ref. [1] specifically focuses on validation of electronic hardware. However, neither of these review studies consider the modelling or direct validation of the functional behaviour of electronic hardware. This is also common to previously mentioned research articles. The present research paper addresses this gap by employing an LSTM-NN model and extending the VSN to a functionality model of an electronic device.

### 2.2. Virtual Sensor Implementation with Neural Networks

A VSN comprises multiple virtual sensors, each of which outputs estimates of a physical quantity based on a numerical model. This model receives inputs of other available and related values, such as operating condition data, sensor readings of alternative physical quantities or at alternative measurement locations. In recent works, DTs and VSNs have implemented the usage of NNs, which has been a recent trend in this field due to the effectiveness of the NN in identifying and modelling the non-linear behaviour in these systems, thus creating even more robust virtual models and allowing for a simpler and cost-effective implementation. Moreover, where there are various sources of data to be analysed, a VSN has been shown to be effective [17] in concurrently utilising these sources in order to infer trends and information about the system that cannot otherwise be seen with traditional signal analysis methods and modelling techniques. One paper [18] proposes a two-phase fault diagnosis method using deep transfer learning where a stacked sparse autoencoder NN is initially trained on a high-fidelity virtual model during the design phase of a manufacturing process, then further trained on the physical process upon inception whilst retaining the information from the designed model to save time and prevent the wasting of knowledge. The authors demonstrate that this technique provides more accuracy than just training on the virtual data at the beginning of the design stage, overcomes any problems with insufficient data during design and can assist in the discovery of any potential design defects before proceeding to a physical implementation. Furthermore, the NN also demonstrates flexibility in adapting to any changes in the working conditions using this method of training.

The VSN method was used to reconstruct accelerometer spectra of physical sensors on rotorcraft from operating conditions and on-board generated statistical values of the sensor readings [19]. The motivation of the study is based on the circumstance that data transfer and storage capabilities are limited in rotorcraft while ample raw sensor spectra are desired for machine diagnostics with more advanced post-processing methods. For autonomous vehicle control systems, virtual sensors provided the vehicle’s planar motion and tyre forces from more cost-efficient physical acceleration sensors [20]. A VSN was applied at Caterpillar in heavy duty machinery with the primary motivation of increasing sensor reliability of degrading emission sensors [7,8]. In the case of a failing physical sensor, the VSN counterpart served to provide backup values and could be used to detect sensor malfunction by monitoring the error between the physical and virtual sensor output. In an analogy with this study, the method presented here compares measured output values against the modelled reference output to identify a faulty or malicious output and employs self-protection features. However, unlike the previously listed studies, the VSN method is extended in this work to not only to estimate sensor values but to model the functional behaviour of an electronic device. Furthermore, an LSTM-NN is utilised as the model foundation, which allows the VSN to consider potential time-dependency in the functional behaviour of the tested electronic device.

### 2.3. Wireless Sensor Network vs. Virtual Sensor Network

Another example of the effectiveness of virtual sensors is demonstrated in [21], where a VSN was implemented as a form of data imputation to ensure that, in the case of a sensor failure in a wireless sensor network (WSN), there would always be useable data to prevent the system from making suboptimal decisions. One thing to note is the clear distinction between the WSN and VSN technologies. In contrast to the VSN, a WSN is a physical network of sensors. They are typically used to monitor environmental conditions and are built on the concept of Internet of Things (IoT), whereby the sensors are wirelessly interconnected and the information gathered from the sensors can be obtained and analysed in real time. A WSN is generally configured towards a specific use case, which sometimes can result in a lack of flexibility in terms of applications. One example of a novel application of WSN is a flood detector system [22], where the authors employ a multi-hop WSN consisting of ultrasonic and water flow sensors to monitor the water levels. A mamdani fuzzy logic system is proposed to process the sensor readings and output flood warnings, with a 96.96% accuracy achieved experimentally. Another recent work [23] implemented a novel localization technique named C-CURVE to localize sensor nodes in a WSN, achieving high accuracy in comparison to other state-of-the-art methods. The monitoring of WSN systems is also a point of interest for researchers. One approach focuses on the monitoring of photovoltaic (PV) systems powering the WSNs. The authors propose the usage of the current and voltage readings of the PV module, battery and load to determine the battery life of the power monitor. Another approach [24] proposes a fault detection and diagnosis system for the sensor nodes in a WSN. An alternative fault detection approach, tailored for use on a self powered sensor network, was proposed in [25]. An SVM-based algorithm was applied on the acceleration and temperature data captured from a machine using the self powered sensor network. The proposed method was able to classify shaker working conditions to an accuracy of 83.6%.

On the other hand, a VSN is a method that can utilise multi-functional WSNs for different use cases. In other words, different types of information can be inferred from the physical WSN, depending on the intended use case. Some examples of VSN-based approaches include sensor fault detection [26]. Using a soft sensing approach, the suspension position of the rear stroke sensor is modelled using a soft sensor NN using a gyroscope, velocity and linear potentiometer sensor readings. The residual of the predicted and real sensor location prediction is then calculated, which feeds into a decision maker that evaluates sensor condition.

A common theme encountered in WSN monitoring methods such as VSNs is the use of machine learning (ML) algorithms for the system modelling and fault diagnosis. One aspect of NN-based methods that is generally overlooked is the assumption of a well trained NN on data that is fully representative of the device’s “normal” working conditions, where normal can be defined as a system output that is expected in that specific context. Whilst in all the aforementioned literature the system model was used to determine if the device is operating as expected, it failed to verify the accuracy of the NN model based on the data used for training. This gap is addressed in this paper with the proposed self-protection tools.

While there is clear promise shown with the usage of NNs for virtual sensors, the field of ML has advanced significantly, with the popularisation of various types of NNs due the significant advantages that they provide in computational power and feature learning, which is further evaluated in the following subsection.

### 2.4. Neural Networks

Many researchers have recently had success using recurrent neural networks (RNN) for their effectiveness in learning the dynamic behaviour of temporal data [27,28], which is useful for the prediction of future data as well as identifying faulty data. This type of NN, however, faces an issue known as the vanishing gradient problem [29] which affects the ability of the NN to train over long periods. The LSTM-NN [30] aims to overcome this issue using a specialised structure with gates that allow for information to be forgotten and replaced, hence overcoming the difficulty faced with training a traditional RNN. The LSTM-NN has been used effectively in various applications [31,32,33] including natural language processing, time series forecasting and fault detection, and has generally exhibited a strong ability to learn long term dependencies. While most of the discussed methods approach learning in a supervised manner, it is expensive and time consuming to label data for the purpose of training a network, especially with larger datasets. The autoencoder [34] was proposed as a method of unsupervised learning. The general concept involves the reconstruction of the input of the network with a dimensionality reduction so that ideally, more features can be identified in the latent space representation. This technique has been used successfully in the context of DTs in [35]. However, unsupervised techniques such as autoencoders and virtual sensors are dependent on the volume of training data and the examples present in the training data, which in many cases may not fully cover the cases encountered by the system in its operational phase. This generally results in unreliable predictions made by NNs, which reduces the integrity of the NN readings and hence decreases the reliability of the NN with accurately modelling system behaviour. These drawbacks are addressed in the proposed method.

### 2.5. Cybersecurity

The motivation of the present study is related to the research field of cybersecurity, which has also embraced ML methods and has seen a steadily increasing interest over the last few years as evidenced by recent review papers [36,37]. These surveys focus on the detection and prevention of computer and network vulnerabilities and attacks based on spam, malware and intrusion detection. Measures for increased security of the software and network system act as supplementary along with the hardware-focused methodology presented in this study, since both target separate system layers. Another branch of cybersecurity research deals with industrial control systems (ICS) and thus puts a stronger emphasis on physical hardware in addition to the cyberspace. A number of review studies covers this space [38,39,40].

Regarding the mitigation of controller attacks, a review study [38] presents four approaches, which concern the control architecture of a Programmable Logic Controller (PLC). The two main distinguished methods are the Trusted Safety Verifier (TSV) [41] and the Controller Controller (C^2^) [42]. The TSV requires a copy of the controller logic and constructs a symbolic logic cycle as the foundation to detect and prevent the execution of a potentially tampered PLC logic. This method has the advantage to detect modifications in advance, but suffers from impractically long execution time when applied to complex systems due to a tree structure of subsequent symbolic cycles, which is often formed in the process. C^2^ is not limited by the system complexity as it relies on a set of engineered safety features, which are used to compare the control signals with the state of the ICS. However, this check is performed at run-time and thus lacks the ability of advance examination provided by TSV. C^2^ intervenes when harmful control signals are detected by either the denial or retry of an operation, PLC notification, or by truncating the command interval to a safe range. These measures aim to mitigate detrimental physical consequences of acceptance or full denial of untrusted commands but are very limited in relation to the potential complexity of the controlled system. For example, truncating of control signals to a safe region can still lead to disastrous consequences when applied contrary to the operators’ instructions or during a critical operating state. This is shown with the case study presented later in Section 4, where a malicious drone controller firmware applies elevon and thrust control signals, which operate in the usual signal range but are designed to provoke a crash.

The secure system simplex architecture (S3A) [43] can be considered as a compromise between TSV and C^2^. S3A requires a more abstract description of the controller process flow compared to TSV and can thus be applied to systems with higher complexity (albeit not with arbitrary high complexity like C^2^). A disadvantage of S3A compared to the proposed VSN is that it relies on the simplex architecture [44] running a so-called safety controller, which takes over operation upon detection of an untrusted state. Compared to the main complex controller, the safety controller runs simplified procedures to merely ensure system stability. However, the design of an appropriate safety controller is associated with extra cost and, depending on the application, a more representative redundant model such as the proposed VSN can be required.

The method of a minimal trusted computing base (TCB) provides a restricted environment, giving only predefined PLC code blocks the control access to physical machinery. This approach is used with the aim to overcome the time constraints of TSV. In this approach, TSV can be applied to these code blocks only instead of the full PLC logic code, increasing its efficiency.

Finally, the semantic security monitoring (SSM) [45] analyses the network traffic of PLCs but follows a related approach to the present study. Different variables, including control and measurement signals are extracted from the Modbus protocol of the PLCs of two operational water treatment plants. These values are constructed into time series, which are used to fit autoregressive (AR) models. In addition, safe control limits are derived from the training data to trigger an alert when they are exceeded. This is done since the AR model is not capable to detect such over range conditions by itself if the exceeding values are reached over a slow progression. This indicates a drawback of this modelling approach compared to the more sophisticated NN models [46]. Another impeding factor is that the method in [45] models each channel individually. In contrast to that, the present study employs a multivariate NN model, which considers potential interrelations between different channels. The study reports limited success, which is attributed to the training data not covering all relevant conditions and false data type classifications between continuous, constant, and attribute data. However, a main benefit of the approach is its cost-effective and straightforward implementation, since a data acquisition through physical access to the monitored devices is replaced by a passive network tap.

Due to the NN foundation of the proposed VSN method, a real time execution similar to C^2^ is achievable, which is a benefit in relation to TSV. Also similar to C^2^, the VSN performs checks and potential interventions during run-time shortly before execution without advance warning. However, all listed alternatives require some degree of knowledge of the PLC logic either in the form of its program code or suitable engineered safety properties. In contrast to that, the proposed VSN methodology constructs a functional model of the controller without the requirement of explicit information on the internal logic. In addition, the presented VSN methodology is not limited to PLCs (such as TSV) and, therefore, can be applied to proprietary embedded controllers or off-the-shelf electronics, greatly increasing its range of applications.

The main features of the discussed methods are summarized and compared to the proposed VSN approach in Table 1. Another factor is the required duration or computational cost for the LSTM-NN training, which can be regarded as a drawback of the proposed method. Detailed evaluations of this aspect are found in [30,47]. However, in relation to other methods, cost and time are saved elsewhere by the VSN approach due to the automatic feature extraction instead of manual safety engineering. In addition, there is no requirement for the development of an additional redundancy system, since it is available from the VSN itself.

### 2.6. Threat to Validity

The search strings in Table 2, used on the Google Scholar and Institute of Electrical and Electronics Engineers (IEEE) repositories, have been provided to show an overview of how the papers for the literature review were obtained, and hence where our current understanding of the current methods in the field was obtained from.

## 3. Proposed Method

This chapter describes the proposed methodology for the identification of the functional integrity of an electronic test device. The first subsection provides an overview of the proposed methodology and the relation between the involved methods. Thereafter, these methods are presented in individual subsections, including the data acquisition, the NN model and self-protection.

### 3.1. Overview

In its original form, a VSN is used to model virtual outputs of physical sensors based on measured outputs from other sensors or physical quantities with related information [7,8]. In the present study, the VSN is implemented with an LSTM-NN and is adapted such that each output signal of an electronic device is assumed as a sensor reading (i.e., VSN output), which is modelled from measurements of the control inputs to the electronic device (i.e., VSN inputs).

**Figure 1:**
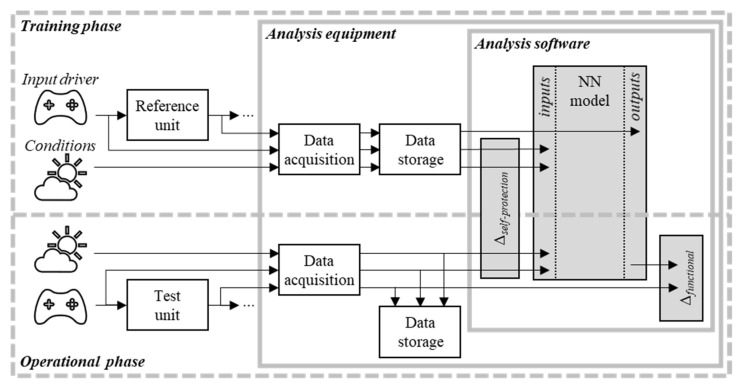
Schematic of the proposed method.

The proposed approach can be subdivided into a training and operational phase, and is illustrated in Figure 1. During the training phase, first, the inputs and outputs of the reference device (i.e., “reference unit” in Figure 1) with proven and trusted functionality are recorded. Additionally, relevant environmental or hardware condition data can be acquired in parallel as illustrated by the “conditions” data stream in Figure 1. The recorded data is used to train a NN, which should adapt the relation between the recorded device inputs (potentially together with the environmental/conditional data) and the desired reference output of this device.

At a later stage, in the operational phase, the same data signals are recorded from an (unknown) test unit. The control input signals of the test unit are then provided as inputs to the trained NN model from the previous step. Since the NN model provides the expected functional reference behaviour, its output is used in a comparison with the test unit. Deviations from this reference baseline (illustrated as “Δfunctional” in Figure 1) indicate potential malfunctioning of the tested device or measurement equipment.

However, such deviations can also arise if the reference behaviour is not accurately replicated by the reference model, e.g., due to insufficient quality or quantity of training data. The reference might not include all input states, operating or environmental conditions. This can lead to previously unseen characteristics of a tested device’s response, which do not necessarily indicate malfunctioning. If the described incomplete reference model is exposed to unknown conditions, the analysis could produce false-positive or generally not meaningful results. To avoid these issues, three methods for self-protection of the proposed analysis are introduced, which are symbolised by the “Δself-protection” block in Figure 1. First, a boundary check is performed, which asserts whether the current inputs are out of bounds compared to the multidimensional input space that is known from the reference training. The resulting true/false statement can indicate, in case of a “false” result, that the conclusion of the analysis should be interpreted with care. Another, more gradual, self-protection check is based on the calculated Mahalanobis distance (MD) between the input-space samples and the currently observed multidimensional operational sample. Finally, a reconstruction of the received inputs is performed by an Autoencoder LSTM-NN. Based on the reconstruction, anomalies in the input measurements (compared to inputs from the training data) are detected.

### 3.2. Data Acquisition

To ensure successful NN training, a sufficiently large data sample is required that covers a wide input range [48]. To learn the behaviour of the device, a known good reference unit has to be recorded in its operation to acquire the operational logs. All input and output ports are probed with the high impedance logger device that performs non-altering signal logging. The logger device is a real-time measurement device running low latency optimized firmware, such that measurements are taken with maximum precision and speed. For this application, the speed is critical to perform the most accurate measurements with minimal time jitter and, therefore, to minimise the distortions of the recorded reference behaviour [49].

The logger device supports measurements in both digital and analogue form. All analogue ports are measured at once with constant time intervals. Digital ports are measured individually by using internal microcontroller interrupts recording the precise time of the digital event. Then all collected measurements are sent to the computer for storage and further processing.

Between logger device controller and computer, there is a limited data transfer bandwidth available [50]. Some of the fast digital events may overwhelm the communication link and interfere with the measurement of the reference device. Therefore, signal compression can be applied if the type of the signal is known. For example, microcontrollers often lack analog output capability and mimic the analog behaviour by switching digital outputs on and off frequently, modulating the duration at which the port is switched on with constant frequency. This method is known as pulse width modulation (PWM). If a PWM signal is detected, then the logger device performs a measurement and logs the duration of the on and off state to calculate the PWM signal value. Similarly, servo signals are digital, but only the duration of switched on time is important, with lower importance set for signal frequency [51]. Servo and PWM signal values are then treated as analog equivalent values as precise switching timing is not a part of device behaviour, unlike discrete digital control signals.

While logging, the reference device has to be powered on and its inputs fed with some signals. There are two possible strategies to feed inputs to the reference device. The first is to feed an input sequence from a previously recorded operational environment, or second, to feed automatically generated inputs. Real operational recorded inputs are usually beneficial for complex systems that require particular input sequences to reach certain states that can be otherwise hard to reach. However, real recorded inputs may not cover the full possible input space during the recorded operation as some edge case scenarios can be exceedingly rare. The automatically generated inputs usually cover most of the input space because input generator functions can reach all boundaries of all inputs, and these functions can do so quickly. However, the generated functions may not represent the expected sequence of inputs and only brute forces the input space.

Acquired raw logs are in mixed raster and vector formats depending on the type of signal. Analogue signals are logged with a constant period, a raster format. Digital signals are logged with the time of a switching event, a vector format that takes minimal storage needed to store the data. Finally, the NN can only accept constant time interval sequential raster data. Therefore, the vector format has to be converted into raster format with a chosen resolution.

### 3.3. Proposed Neural Network Model

**Figure 2:**
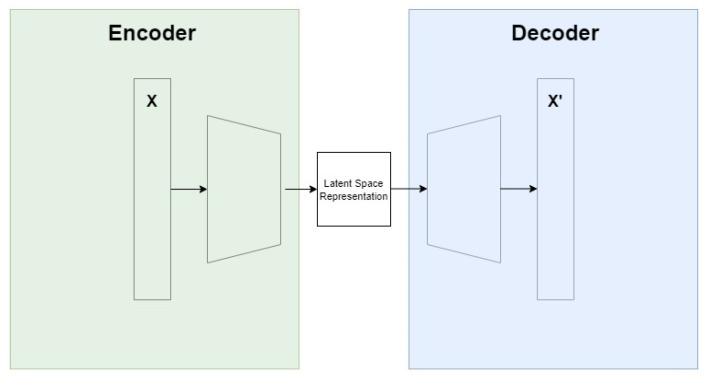
Visualisation of autoencoder operation, modified from [34].

The NN model being proposed for use as a VSN is a Long-Short-Term-Memory (LSTM) autoencoder. The purpose of this architecture is to reconstruct the raw input data with a dimensionality reduction so that new features in the latent space representation of the data can be identified and learnt by the NN. A visualisation of this is shown in Figure 2, and a formal definition is provided in Equations (1) and (2), courtesy of [34].

(1) f(x)=latent space.

(2) g(f(x))=x’

#### Complete Neural Network Architecture

**Figure 3:**
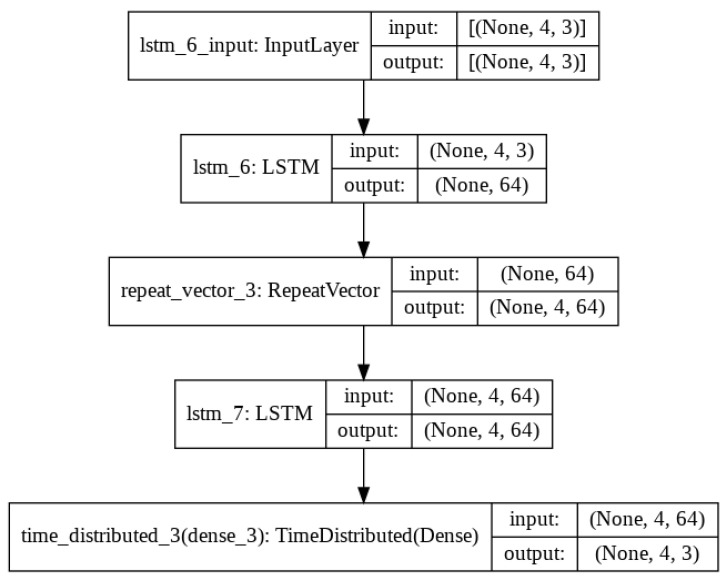
Proposed long-short-term-memory (LSTM) autoencoder model.

The proposed architecture used for reconstructing the signal is illustrated in Figure 3. The data are first passed through an LSTM layer where the dimensionality reduction is performed. The RepeatVector layer then reshapes the data into vector format so that the subsequent LSTM layer can be applied to decode the information. A TimeDistributed layer is used as the output layer to apply a dense layer to each temporal slice of its input, which produces the final reconstruction of the input data from each signal collectively. Tensorflow [52] was used for the compiling and training of the NN.

### 3.4. Data Pre-Processing Stage

Although the data are used in its raw temporal format, Z-score normalisation is applied to boost the efficiency and speed of training without impacting the shape and features of the data. This method of normalisation is advantageous in comparison to min-max normalisation when used on data with significantly outlying samples since when anomalies are present, min-max normalisation will result in a tight grouping of the non-outlying data, which will impact the ability of the NN to learn the data features effectively. This operation was performed using Equation (3):

(3) $$z=\frac{\left(X\;-\;\mu \right)}{\sigma }$$

where X is a non-normalised data point, μ is the mean of a subset, σ is the standard deviation of a subset and z is a normalised data point.

### 3.5. Network Training and Threshold Calculation

For hyperparameter selection, each variable was individually iterated with the value giving the best validation loss and mean squared error (MSE) of accuracy on the test set used to tune the next hyperparameter until all hyperparameters were tuned. The validation loss was observed to ensure that the NN has optimal generalization ability, and the MSE, which is the mean of the square of the difference between the real and predicted values, was used to gauge the consistency of the predictions. The result of this experiment is shown in Table 3. It is worth noting that overfitting was minimal, even before the tuning of the hyperparameters to optimal values. This was mainly due to the simplicity of the data as well as its consistency.

### 3.6. Self-Protection

If the difference between currently observed operational measurements and the predicted output of the proposed NN model exceeds a threshold, a potential change in the input/output behaviour of the tested or monitored device or measurement equipment is indicated. In these cases, unexpected changes, e.g., due to degrading hardware or modified firmware, can be detected and addressed in a timely manner as intendent. However, the representation of a real-world system by a model such as the proposed NN is an approximation and thus includes prediction errors. These modelling errors are another potential source for a mismatch between observed and predicted data samples. Errors in NN outputs can commonly occur due to insufficient training data [47]. Such insufficient training data might not be representative of potentially rare operational conditions, i.e., the operational input/output space is not contained within the training data space. Even when this condition is satisfied, an insufficient quantity of training data can also lead to model errors.

To address this issue and be able to distinguish between functional changes in the actual hardware or sensors on the one hand and model errors on the other, the proposed methodology implements the three following indicators, i.e., self-protection features.

#### 3.6.1. Self-Protection 1: Multidimensional Boundary Check

The first indicator is a boundary check, where the currently observed samples are compared to the boundaries of the training data input space. If, during operation, an observed input feature quantity falls below the minimum or exceeds the maximum value of that feature’s set of training samples, this operational sample Is flagged. Such multidimensional out-of-boundary sample represents an operating state, which is essentially previously unseen by the model and as such the corresponding NN should be interpreted with care.

#### 3.6.2. Self-Protection 2: Mahalanobis Distance

The second indicator for potential model errors is calculated as the MD between the currently observed multidimensional input sample and the multidimensional training input data space as per Equation (4).

(4) $$D_{\mathrm{MD}}\left(x\right)=\sqrt{\;{\left(x\;-{\;\mu }_{y}\right)}^{T}{\;K}_{\mathrm{yy}}^{-1}\;\left(x\;-\mu_{y}\right)},$$

where x is the operational input sample vector, which contains a single sample of the measured model input features, μ_y_ is the vector of training data sample means of each feature and K_yy_ is the covariance matrix of the training data samples.

As the MD is given in standard deviations, it is a continuous metric in contrast to the binary boundary-check and is also affected by the quantity of used training samples, thus offering a deeper insight into the certainty of the model prediction. Consequentially, for higher MD values of an operational sample the corresponding model predictions are assumed to be less trustworthy.

#### 3.6.3. Self-Protection 3: Input Signal Reconstruction

The third proposed self-protection tool is based on the reconstruction of each input signal from the measured operational inputs. This is achieved by a NN, which is trained with device input measurements both on the NN input and output for reconstruction. The NN architecture used is otherwise identical to that presented in Section 3.3. During operation, any reconstructed input values straying from the input space in the training set is identified and flagged as an anomaly, where an anomaly is defined as a datapoint or set of datapoints that do not follow the expected trend of the data based on the given inputs. While this may not be due to a fault of the tested device, the operator will be informed so that they can observe the subsequent outputs from the device. Any abnormalities then detected in the output are more likely to indicate an unexpected change in the operating conditions rather than a malfunction of the device.

An indication of an unseen input condition is determined using a threshold on the error residual, determined from the reconstruction accuracy from the validation set. Any reconstructed values exceeding this threshold are likely to be outside the trained input space, which is suitable for reliable performance of the main NN introduced in Section 2.1.

## 4. Experimental Demonstration with a Remote-Controlled Drone

**Figure 4:**
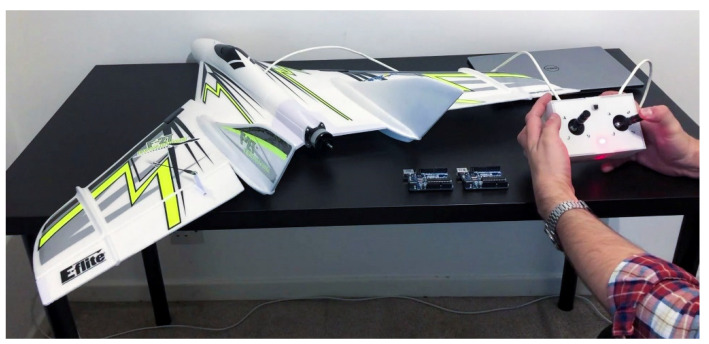
Remote-controlled drone setup.

A remote-control drone (Figure 4) is chosen as an illustrative example to demonstrate how a compromised drone’s onboard actuator mapping could cause a severe issue to the flight ability and safety of the drone. The drone is a flying wing type drone with one motor and two elevons. Elevons and the motor are controlled by an onboard radio receiver and a controller with programmable actuator mappings. Usually, these controllers are benign plug-and-play devices that come with programmed default actuator mappings suitable for the drone. However, in the case of malicious or negligent actuator mapping, changes in the critical modes of operation may be catastrophic and cause a crash.

### 4.1. Measurement Setup

The drone’s onboard controller takes three analogue inputs of pitch $R_{y}$ (input signal 1), roll $R_{x}$ (input signal 2), throttle T (input signal 3) and gives three servo outputs signals for right elevon $E_{r}$ (output signal 1), left elevon $E_{l}$ (output signal 2), motor power $M_{1}$ (output signal 3). The throttle controls the motor power. Roll input is mapped for both elevons to actuate in opposite directions. Pitch input controls both elevons in the same direction and controls additional power to the motor when the drone flies up.

(5) $$E_{r}=\frac{R_{y}-R_{x}}{2}$$

(6) $$E_{l}=\frac{R_{y}+R_{x}}{2}\;$$

(7) $$M_{1}=T\;+\frac{R_{y}}{2}\;$$

Standard actuator mappings are known to us. However, neither the logger device nor any part of the NN training process are concerned with the actuator mapping formulas. This allows the system to remain impartial of the test device, and the methodology remains valid for arbitrary test devices.

### 4.2. Experimental Results

Experiments were run on a computer with an Intel i7-8750H processor, NVIDIA GeForce GTX 1070 graphics processing unit (GPU), 16 GB memory and Windows 10 Operating System.

#### 4.2.1. Neural Network Training and Threshold Calculation

The data used for training and testing consists of: a 5-min subset of input and output data from the reference device used as training data, a 30 s subset of input and output data from the reference device used as validation data and a 30 s subset of input and output data from the test device, with faults induced by malicious code, used as test data.

For the first experiment, to test the multidimensional boundary check and MD self protection features, the NN will be trained with the full pitch, roll and throttle input data from the reference device as the NN input and the expected output as the right elevon, left elevon and the motor power (Section 4.2.2). The mean absolute error (MAE) of the worst training prediction for each signal will be used as the threshold MAE values, for which any subsequent prediction found to exceed these MAE values is defined as an anomaly and is flagged as such. The NN and self protection features will then be tested on the same signals on the test device with the malicious code (Section 4.2.3).

The second experiment will reduce the training data to a 5 s subset of the full dataset to simulate a training set that is not fully representative of the range of data in the expected input space during device operation (Section 4.2.4). Similar to the previous experiment, the worst training prediction MAE for each signal will be used as the threshold MAE values. The NN will then be tested on the same test data as the previous experiment for anomalies.

The NN used for the final experiment will be trained to reconstruct the device input data using the full reference device input dataset (Section 4.2.5). The worst training prediction MAEs will be used as threshold values for the test with artificial bias faults inserted into the data. Any datapoint exceeding the value of the threshold MAE of the respective signal is flagged as an anomaly.

A comparison of the training results for each experiment is shown in Table 4. Table 5 shows a comparison of the threshold values calculated for each NN. For reference, the range of encountered data for each signal is also shown in Table 6.

#### 4.2.2. Input-Output Mapping with Residual Error Threshold

Using the optimal hyperparameters in Table 3, the model was trained on a 5-min subset of the data from the reference device, validated on a 30 s subset from the reference device and tested on a 30 s subset of the test device with faults inserted. The results in Table 4, Experiment 1 show the summarised results for this case.

**Figure 5:**
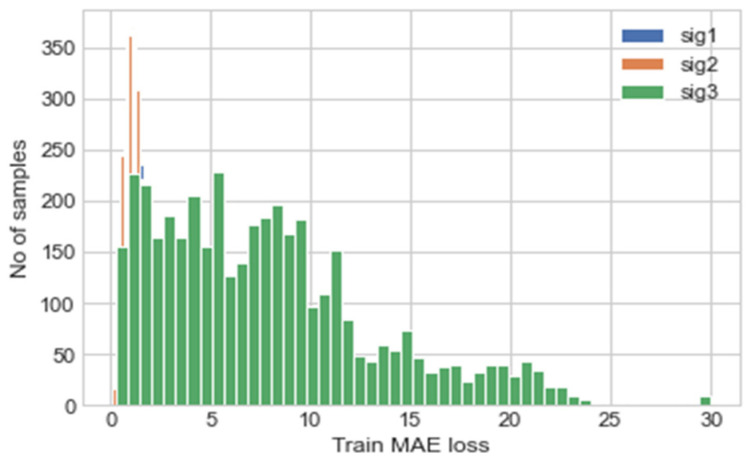
Histogram depicting MAE of each prediction for each input/output channel, where sig 1, sig 2 and sig 3 represent the right elevon, left elevon and motor power respectively.

Using the trained model, the validation data was predicted, and the MAE of the predictions are calculated; this is illustrated using a histogram in Figure 5. The maximum MAE values for each signal are used as the thresholds for the anomaly detection, these are shown in Table 5, Experiment 1. The MAE values are calculated using the normalised data values and not the actual values, hence the magnitude of the values calculated.

#### 4.2.3. Virtual Sensor Network (VSN) Testing

**Figure 6:**
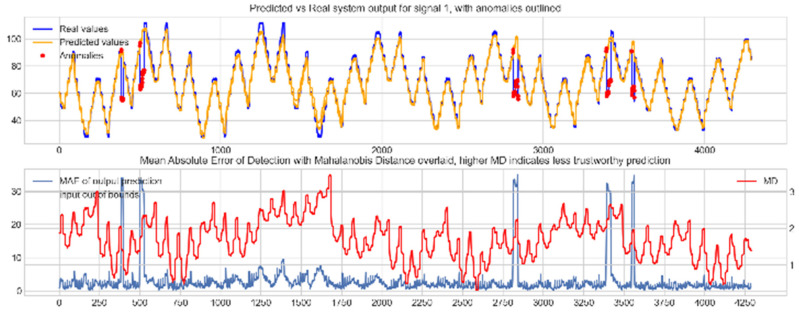
Predicted vs. Real NN prediction for output signal 1, the right elevon.

**Figure 7:**
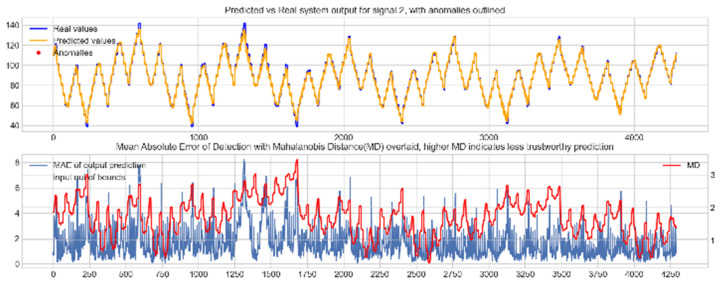
Predicted vs. Real NN prediction for output signal 2, the left elevon.

**Figure 8:**
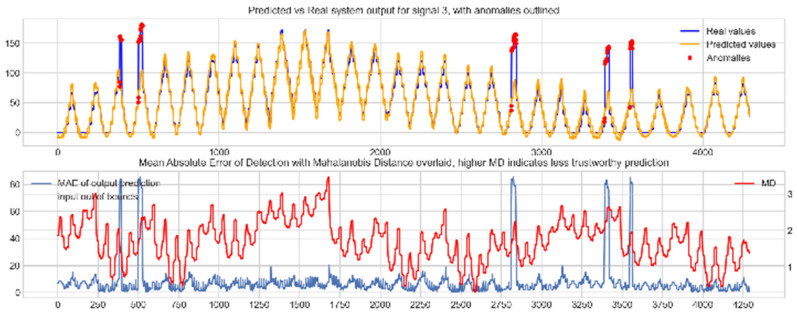
Predicted vs. real NN prediction for output signal 3, motor power.

Input data from the test device was then used to test the mapping ability of the NN. The data from the three drone input signals were input into the NN; Figure 6, Figure 7 and Figure 8 show the prediction of the NN in comparison to the actual data values in the top subplot, and any anomalies found in the data highlighted in red. The MD, MAE and boundary check are plotted in the bottom subplot, where the blue line represents the MAE on the left-hand scale, and the red line represents the MD on the right-hand scale. Any yellow shaded area represents a datapoint outside the expected input space.

#### 4.2.4. Boundary Check and Mahalanobis Distance Self-Protection Testing

**Figure 9:**
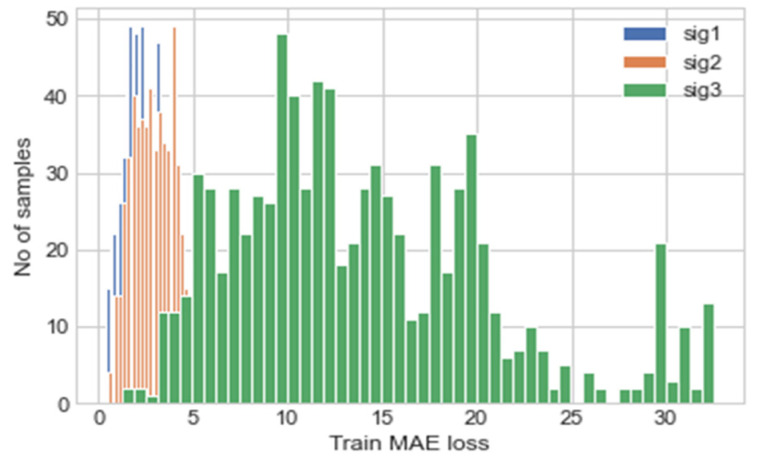
Histogram depicting MAE of each prediction for each input/output channel.

To test the effectiveness of the proposed self-protection features, the model was trained on a 5 s subset of the training set acquired from the reference device, instead of the full 600 s. This will decrease the accuracy of the NN predictions and result in the input data from the test device being outside the input space expected by the NN, thus causing an unreliable NN prediction. Table 4, Experiment 2 depicts the results of the training, Figure 9 the MAE of the validation data predictions, and Table 5, Experiment 2 the calculated MAE threshold from the validation data.

**Figure 10:**
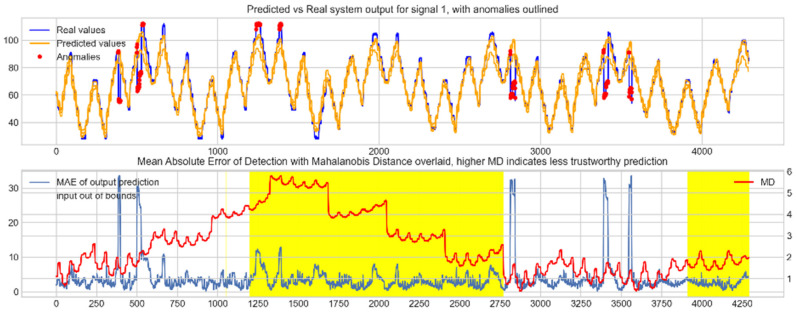
Predicted vs. real device output for signal 1, the right elevon, with anomalies labelled (**top**); MAE, MD and boundary check corresponding with the prediction (**bottom**). The yellow shaded areas represent new inputs outside expected input space.

**Figure 11:**
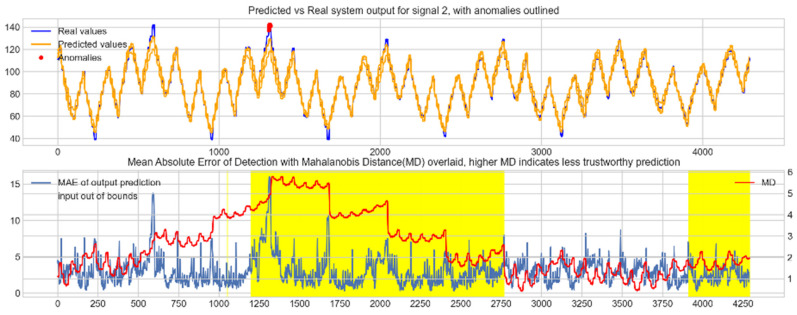
Predicted vs. real device output for signal 2, the left elevon, with anomalies labelled (**top**); MAE, MD and boundary check corresponding with the prediction (**bottom**). The yellow shaded areas represent new inputs outside expected input space.

**Figure 12:**
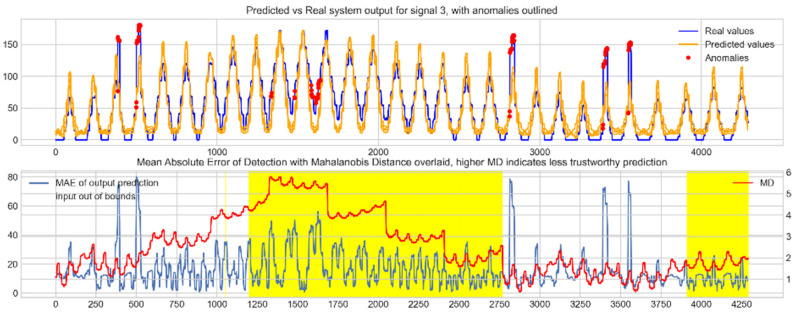
Predicted vs. real device output for signal 3, the motor power, with anomalies labelled (**top**); MAE, MD and boundary check corresponding with the prediction (**bottom**). The yellow shaded areas represent new inputs outside expected input space.

With these threshold values, the NN was tested with the reconstruction of the input test device data. The boundary check and MD between the training input data and the test input data were also calculated and superimposed over the MAE plot. Figure 10, Figure 11 and Figure 12 show the results of the described plots. The figures follow a similar layout to Figure 6, Figure 7 and Figure 8.

#### 4.2.5. Self-Protection Feature 3—Input Reconstruction

**Figure 13:**
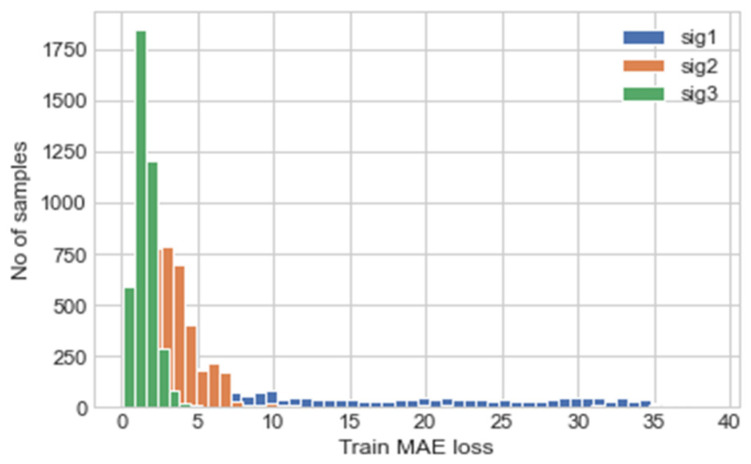
Histogram depicting MAE of each prediction for each input/output channel.

The NN model in Figure 3 with the hyperparameters shown in Table 3 was used for the input signal self-protection. The model was trained on the same 5-min subset from reference device previously used, validated on the same 30 s subset from the same device and tested on the 30 s subset from the test device. The results of the training are shown in Experiment 3. The MAE of prediction for the validation set was then calculated and illustrated, this is shown in Figure 13. The threshold values for the anomaly detection was determined using the maximum MAE value from the validation prediction and are shown in Table 5, Experiment 3.

#### 4.2.6. Reconstruction Testing

**Figure 14:**
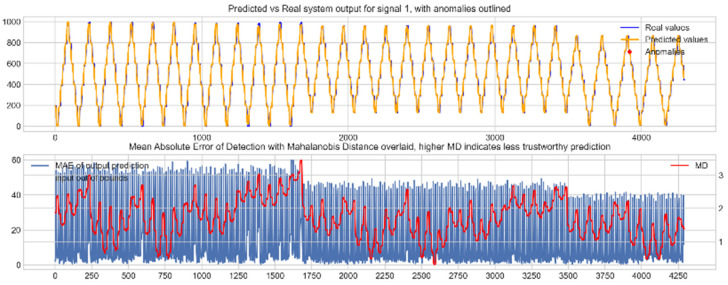
Predicted vs. real device input for signal 1, the right elevon, with anomalies labelled (**top**); MAE, MD and boundary check corresponding with the prediction (**bottom**).

**Figure 15:**
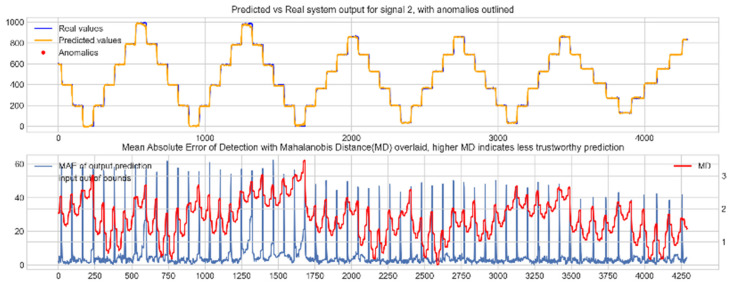
Predicted vs. real device input for signal 2, the left elevon, with anomalies labelled (**top**); MAE, MD and boundary check corresponding with the prediction (**bottom**).

**Figure 16:**
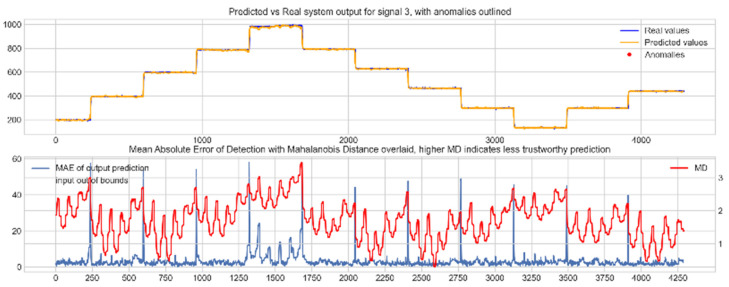
Predicted vs. real device input for signal 3, the motor power, with anomalies labelled (**top**); MAE, MD and boundary check corresponding with the prediction (**bottom**).

The reconstruction ability of the NN was then tested. The results of the predictions for each input signal are shown in Figure 14, Figure 15 and Figure 16, where the top plot represents the real vs. predicted input reconstruction and any anomalies highlighted in red and the bottom plot represents the MAE on the left-hand scale and MD on the right hand scale.

**Figure 17:**
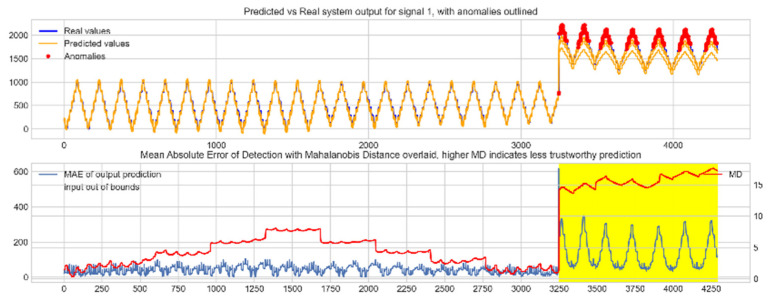
Bias fault inserted in input signal 1, the right elevon, from data point 3250 onwards, with NN anomaly detection highlighted in red and inputs outside the expected input space shaded in yellow.

**Figure 18:**
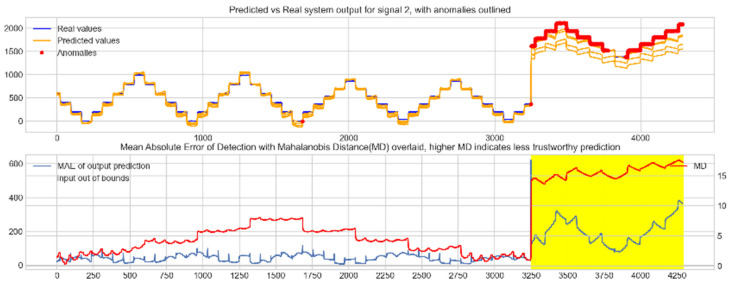
Bias fault inserted in input signal 2, the left elevon, from data point 3250 onwards, with NN anomaly detection highlighted in red and inputs outside the expected input space shaded in yellow.

**Figure 19:**
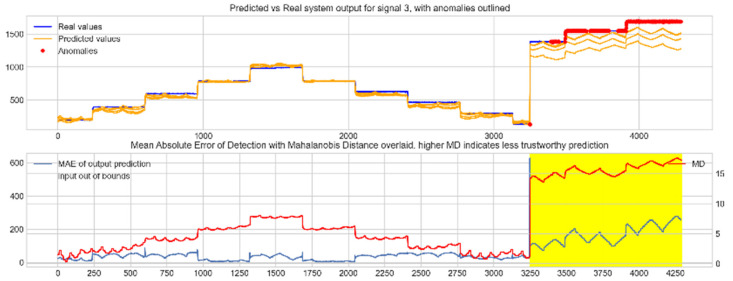
Bias fault inserted in input signal 3, the motor power, from data point 3250 onwards, with NN anomaly detection highlighted in red and inputs outside the expected input space shaded in yellow.

A bias fault was then artificially inserted in each input signal in the test data from data point 3250 onwards, to simulate test data lying outside the expected input space of the device. Figure 17, Figure 18 and Figure 19 show the NN detecting and flagging the outlying data points, marked in red on the top plot, and the respective MAEs, MDs and boundary checks plotted on the bottom plot in each figure.

## 5. Discussion

The results in Figure 6, Figure 7 and Figure 8 show the input-output mapping ability of the proposed NN with sufficient testing data. In each Figure, the top subplot represents the real test device output values of the elevons and motor power vs. the NNs predicted output based on the drone input of the pitch, roll and throttle. Any prediction MAE exceeding the threshold values set in Table 5 is highlighted in red and is thus outlined as an anomaly. The results show that the NN is able to identify the anomalous data accurately and robustly on the datapoints clearly outlying from the expected data trend, which are predetermined anomalies induced by malicious code implanted in the drone controls. Furthermore, with sufficient data, the self-protection features do not give any indication of an unreliable prediction, with all the test data being in the expected input space, represented by the lack of any yellow shaded areas and the MD staying consistently low, shown in the bottom plot in each figure.

When training the NN with incomplete input data that was not fully representative of the input space to be expected from the device operation, the self-protection features were effective in highlighting the unreliable measurements. For instance, on Figure 11, data point 1300 is highlighted as an anomaly by the NN, however when taking into account the MD, which is at a maximum at that point in relation to the lower values experienced with the previous experiment with the full input training data, and the boundary check, which flags the data as being outside the input space, represented by the highlighted yellow area, this anomalous prediction should not be trusted. On the other hand, the anomalies flagged in the previous experiment are still labelled as anomalies with a small training set, and with MD at a similar level to the level observed with a full training set and the input data being inside the input space boundary represented by a lack of yellow shading, the predictions can be regarded as more reliable and so, therefore, these anomalous datapoints can be further investigated by the operator.

The input self-protection NN was also effective in identifying abnormal trends in the input data. With a full training set of drone input data and no artificial faults inserted in the data, the results in Figure 14, Figure 15 and Figure 16 indicate the input reconstruction followed the expected trend of the input data well, shown by the matching of both plots in the top plot in each figure and did not flag any outlying data in red. However, with the bias fault inserted into the data from data point 3250 onwards, the results in Figure 17, Figure 18 and Figure 19 show the NN clearly and effectively identifying the changepoint as well as the majority of the outlying data in red. Furthermore, the bottom plot indicates by the out of bounds boundary check self-protection tool in yellow that these datapoints are outside the expected input space, signifying to the operator that the input data is not encountered, and the MAE can be clearly seen to increase past datapoint 3250.

The proposed methodology provides a major benefit over existing alternatives covered in the literature review (Section 2.5) in the sense that it does not require advance knowledge about behaviour of the target electronic device and is not limited to the application to PLCs like TSV. This is due to the fact that the device behaviour can be modelled by the proposed VSN approach as long as the device inputs can be driven while their values are logged along with the resulting device outputs. In spite of the number of drawbacks of TSV, it has the advantage over other methods (including the proposed VSN) that it is able to evaluate the full PLC code in advance. However, since the proposed VSN model is based on an LSTM-NN, it provides both a fast execution time (in contrast to TSV) while being capable of representing complex functional systems with non-linearities and time-dependency. Therefore, the VSN control model representation can act as a full fault-redundant signal source, in contrast to the rather limited intervention measures of C^2^ and S3A. To the best of the authors’ knowledge previous published studies in the domains of DT and VSN either model a physical condition of devices and machines or infer virtual sensor values from alternative data sources. However, they do not model and analyse the functional behaviour of electronic devices like demonstrated in this study. Furthermore, the reviewed body of research in these domains appears to assume correct model representations, while the present work suggests the detection of potential model errors with the presented self-protection methods to further increase system reliability.

### 5.1. Limitations

Despite a number of advantages of the proposed method listed above, there are certain limitations associated with it. One aspect that is difficult to determine, and can therefore be seen as a limitation, is the certainty of the functional integrity of the reference device. Whilst there are existing techniques for determining the reliability of an electronic device, training a NN on a reference device, which is itself faulty or exhibits undesired behaviour, could cause major issues with the reliability of the model. Furthermore, the self-protection features operate based on the data from the reference device, rendering them unable to address this issue as the features would not flag any faulty or unreliable data that was seen as normal on the faulty reference device.

NN based virtual sensing is bottlenecked by the modelling ability of the NN, which is generally determined by the layer depth and hence the number of trainable parameters present. When utilising this framework of functional integrity checking, the NN model used for mapping and input reconstruction must be designed and implemented with the complexity of the data and feature richness in mind. Failing to account for this will cause baseline predictions to be unreliable and would reduce the overall sensitivity of the NN with anomaly detection. This is heavily detrimental to the overall ability of the system, as there may be some less significant anomalies that the NN would fail to spot as a result of this issue.

For optimal performance, the full range of relevant operational conditions and input signals of the reference device should be covered in the training phase (Figure 1). However, this limitation is mitigated by the presented self-protection features, which aim to identify such conditions where increased modelling errors are expected.

As discussed in the literature review (Section 2.5), the proposed method is applied at run-time to perform potential warnings or interventions directly before a faulty or malicious response of the monitored device is passed on to other system components. This circumstance is less relevant in a product development and manufacturing phase. However, when deployed in an operational setting, this requires an application-dependent fail-safe logic to handle the triggered warning in real-time, e.g., by substituting the identified faulty response by the virtual reference response (in case of a high fault-certainty indicated by self-protection features) or by providing a user warning otherwise.

Finally, the integration of the proposed VSN method into a complete system structure for deployment is outside of the scope of this paper and has not been fully developed at this stage. An important factor to consider for this task is the protection of the VSN software and hardware against manipulation itself. Existing cybersecurity approaches, such as the implementation of the proposed VSN method within separate and non-writable hardware, can be utilized for this purpose.

## 6. Conclusions and Future Work

The present study introduced a methodology for the identification of the functional integrity of electronic devices based on an NN model of a reference device unit. The methodology was applied to actual measurements of drone hardware with desired controller response on one hand and a malfunctioning controller on the other hand. The results of this experiment demonstrate the effective application of the proposed methodology as discussed in the previous chapter.

In addition, the scenario of an insufficiently trained NN was examined to investigate the performance of self-protection features, aiming to distinguish false model outputs from actual changes in the measured device behaviour compared to the reference device. Three independent self-protection features with increasing complexity were implemented and applied to both types of NN models (with either complete or insufficient training). The results obtained show that the output of the self-protection tools correlates well with the MAE of the model output in areas where the increased MAE is due to model errors. Therefore, the self-protection methods successfully indicated modelling errors, which can likely remain undetected otherwise.

Concrete examples for relevant application areas of the presented methodology include the monitoring, diagnosis and redundancy of electronic controller functionality of systems such as aircraft, vehicles, industrial machinery, power plants, automated production lines and robotics in general. On a broader scope, the presented methodology has applications in the validation of the behaviour of tested device units during prototyping and manufacturing. In a global economy, electronic hardware components are typically acquired from external suppliers or as off-the-shelf products due to cost-efficiency or the incapability of in-house production. However, the manufacturing quality, processes, and potential changes thereof are often not transparent to the customer in these cases. Validation of the functional integrity of electronic hardware by the proposed VSN approach combats the risk associated with the hardware acquisition from external suppliers. During the operational phase, the methodology can be used for monitoring as a foundation for predictive maintenance and to prevent potential consequential hardware damage, downtime and risk of injury. Finally, the virtual model response can be used as a substitute during operation to bypass faulty behaviour of the device, i.e., as a means of intervention by redundant hardware or failure logic. This is especially relevant to safety-critical applications, which at the same time demand high certainty in decision-making. The presented self-protection features can be used to address this issue by reducing the risk of false interpretations due to model errors. Since the self-protection methods identify specific conditions where the used NN model shows flaws, they also aid a targeted improvement of the NN with the identification of supplementary training data.

Although an experimental validation of the proposed methodology is given in the present study, for future work it is suggested to evaluate the methodology in additional case studies and in-field operational applications. While the case study in this paper presented a case with malfunctioning firmware, subsequent research could evaluate the method on faults originating from hardware defects. Electronic components such as resistors or capacitors on controller hardware could be artificially removed or damaged for this purpose. In the future, alternative NN architectures can be implemented, and the presented self-protection methods can be compared in more detail to investigate their performance in specific applications. Finally, the identified current limitations, which are discussed in Section 5.1, can be addressed in future work for further development of the method.

## Figures and Tables

**Table 1:** Comparison of related methods.

Method	Typical Detection Time ^1^	Supported System Complexity	Intervention Mechanics	Automatic Feature Definition	Indication of False Positives	Detection in Advance
S3A [43]	5.7 μs	medium	Limited logic provided by safety controller	No(Manually engineered)	No	No
C^2^ [42]	0.1–0.2 ms	high	Limited (denial, retry, notification, command interval truncation)	No (Manually engineered)	No	No
TSV on minimal TCB [41]	0.1–120 s (Complexity-dependent)	medium	None (Detection only)	No (Manually engineered)	No	Yes
SSM [45]	N/A	medium	None (Detection only)	Yes (Detection from the AR reconstruction error)	No	No
VSN (this paper)	0.14 ms	high	Full functional redundancy by VSN output	Yes (Detection from the NN reconstruction error)	Yes (By self-protection features)	No

^1^ Indicative values, since the execution time is obtained on different hardware and different case studies used in the respective papers.

**Table 2:** Search strings and databases searched on for the literature review.

Search String	Database
Virtual sensor network	Google Scholar, IEEE
Virtual controller	Google Scholar
Digital twin controller	Google Scholar
Electronics digital twin	Google Scholar
Virtual sensing	Google Scholar, IEEE
Soft sensing	Google Scholar, IEEE
Wireless sensor network	Google Scholar
Digital twin neural network	Google Scholar
Virtual Sensor Neural Network	Google Scholar, IEEE
Controller Cybersecurity	Google Scholar, IEEE

**Table 3:** Optimal hyperparameters found for this task.

Hyperparameter	Value
The number of epochs	100
Optimiser	Nadam
Loss function	Huber
Learning rate	0.001
Time steps (lookback)	4
Hidden layer width	64
LSTM activation	tanh
Batch size	1000

**Table 4:** Comparison of training results for the input/output mapping neural network (NN) on the full dataset, the input/output mapping NN on the reduced dataset and the input reconstruction NN; where avg is shorthand for average.

Experiment	Trainable Parameters	Avg Training Loss Mean	Avg Validation Loss Mean	Avg Prediction Mean Squared Error (MSE)	Avg Training Time (s)
Experiment 1: Full input data for output data prediction training	50,627	0.0013	0.0018	1.19	34.7
Experiment 2: Reduced input data for output data prediction training	50,627	0.0138	0.0338	97.80	23.6
Experiment 3: Full input data for input reconstruction training	50,627	4.2598 × 10^−4^	5.5175 × 10^−4^	0.47	40.1

**Table 5:** Comparison of mean absolute error (MAE) threshold values used in each experiment, where the experiments have the same labels as Table 3.

Signal	Prediction MAE threshold		
	Experiment 1	Experiment 2	Experiment 3
Right elevon	9.26	12.81	67.64
Left elevon	10.10	15.01	10.10
Motor power	44.92	48.73	44.92

**Table 6:** Range of values for the input and output signals in each of the training, validation and testing datasets respectively.

		Training Data			Validation Data			Testing Data		
		sig 1	sig 2	sig 3	sig 1	sig 2	sig 3	sig 1	sig 2	sig 3
Input Data	Minimum Value	0	0	0	0	8	97	0	0	122
	Maximum Value	995	998	1003	979	927	455	994	996	998
Output Data	Minimum Value	19	39	0	19	47	0	28	39	0
	Maximum Value	119	143	173	116	138	88	112	142	181

## Data Availability

Not applicable.
